# Making a Dent in Dent Disease

**DOI:** 10.1093/function/zqaa017

**Published:** 2020-09-11

**Authors:** Katherine E Shipman, Ora A Weisz

**Affiliations:** Renal-Electrolyte Division, Department of Medicine, University of Pittsburgh School of Medicine, Pittsburgh, PA 15213, USA

**Keywords:** tubular proteinuria, Dent disease, endocytosis, recycling, megalin, cubilin

## Abstract

Dent disease (DD) is a rare kidney disorder caused by mutations in the Cl^−^/H^+^ exchanger ClC-5. Extensive physiologic characterization of the transporter has begun to illuminate its role in endosomal ion homeostasis. Nevertheless, we have yet to understand how loss of ClC-5 function in the kidney proximal tubule impairs membrane traffic of megalin and cubilin receptors to cause the low molecular weight proteinuria characteristic of DD. This review identifies open questions that remain to be answered, evaluates the current literature addressing these questions, and suggests new testable models that may link loss of ClC-5 function to tubular proteinuria in DD.

Normally, proteins that escape the glomerular filtration barrier are efficiently recovered by the proximal tubule (PT), and the apical endocytic pathway of PT cells is uniquely specialized to accommodate the uptake of a broad range of filtered ligands. Protein recovery occurs through endocytosis mediated by the multi-ligand receptors megalin and cubilin.^1^ These receptors are constitutively internalized in clathrin-coated pits (CCPs) into small apical endocytic endosomes (AEEs) that fuse with large apical vacuoles (AVs). The soluble contents of vacuoles are delivered to lysosomes, whereas membrane constituents such as megalin and cubilin receptors are recycled to the apical surface. Recycling occurs from both AEEs and AVs via tubular structures termed dense apical tubules (DATs) that are abundantly present in the subapical cytoplasm of PT cells^2^^,^^3^. Nielsen demonstrated two-phase membrane recycling in PTs isolated from rabbits with the initial, rapid phase occurring from smaller AEEs and the later phase from large AVs.^3^ While the Rab proteins and trafficking machinery involved in either phase remain unknown, in accordance with general membrane trafficking terminology, recycling from early (AEE) versus later (AV) compartments along the endocytic pathway is termed fast recycling and slow recycling, respectively.

Dent disease (DD) is a rare, X-linked disorder primarily resulting from the loss of function of the voltage-gated chloride/proton exchanger ClC-5, which presents with low molecular weight (LMW) proteinuria and frequently progresses to chronic kidney disease and end-stage renal disease.^4^^,^^5^ In addition, some cases of DD (termed Dent disease 2) have been traced to a subset of mutations in the phosphatidylinositol 5-phosphatase OCRL, which can also cause the more severe disease Lowe syndrome.^6^ Currently, over 200 different pathogenic variants of *CLCN5* have been described, the majority of which result in missense or frameshift mutations with many resulting in truncation of the protein.^7^^,^^8^ Most of the identified variants have not been functionally studied and even fewer have been studied in representative kidney cell lines or tissue. While all DD patients exhibit LMW proteinuria, the presence of other symptoms and severity of all symptoms vary greatly among patients.^9^ Variability in disease severity may be in part due to the differing degree of residual ClC-5 function of different variants. Knockout (KO) of ClC-5 in mice largely phenocopies the human disease and results in LMW proteinuria. These mouse models have reduced uptake of filtered proteins as well as a decrease in total megalin and cubilin expression without reported changes in mRNA.^10–12^ The LMW proteinuria observed in DD mouse models is attributed to the reduced expression of endocytic receptors. This reduced expression of megalin and cubilin could also play a role in other symptoms of DD such as hypercalciuria, nephrocalcinosis, and nephrolithiasis, because these receptors internalize a number of hormones and vitamin carrier proteins, including parathyroid hormone and vitamin D binding protein, that regulate calcium homeostasis.^1^^,^^13^

Since mutations in ClC-5 were first identified as the cause of DD, we have developed a greater understanding of the physiologic role of ClC-5 in endosomal ion homeostasis. Despite this, how loss of function of ClC-5 results in decreased megalin and cubilin expression to cause LMW proteinuria remains elusive. Specifically, there has been little consideration given to how alterations in endosomal pH or chloride content translate mechanistically to loss of megalin and cubilin receptors. This is particularly a puzzling question because impaired acidification along the endocytic pathway typically leads to protein stabilization rather than loss. This review focuses on the interface between the cell biological and physiological approaches needed to determine the function of ClC-5 in the proximal tubule and to understand how trafficking goes awry in its absence. We posit critical issues that remain to be addressed, describe the data to date and the limitations of these studies, and suggest testable hypotheses to resolve the many open questions in the field.

## Role of ClC-5 in PT Endosome Ion Homeostasis

ClC-5 activity is clearly required to maintain proper endosomal pH and [Cl^−^] in the PT. However, we still do not fully understand whether it is the changes in endosomal [Cl^−^] and/or [H^+^] in PT cells lacking functional ClC-5 that result in aberrant trafficking of megalin and cubilin. Moreover, it remains unclear how endosome maturation and cargo sorting are impaired by defective ion homeostasis in DD to result in reduced levels of megalin and cubilin.

**Figure 1.**
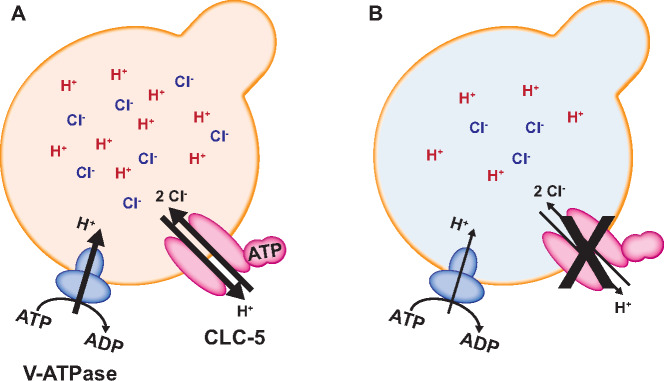
Role of ClC-5 in Endosomal Ion Homeostasis. (**A**) ClC-5 is colocalized with V-ATPase in early endosomal compartments. ClC-5 is a Cl^-^/H^+^ antiporter that exchanges two Cl^−^ ions for one H^+^. An endosomal [Cl−] pool is necessary to maintain the import of H^+^ by V-ATPase and maintain endosomal pH. ATP binding to the CBS domains of ClC-5 enhances its transport activity and may facilitate functional coupling between ClC-5 and the V-ATPase. (**B**) Loss or reduction of ClC-5 function in DD causes a decrease in endosomal [Cl^−^] and impairs endosomal acidification. Whether the change in endosomal pH contributes to the pathology of DD remains unclear (see text).

ClC-5 is largely co-localized with vacuolar ATPase (V-ATPase) in kidney cells,^14^^,^^15^ but how it modulates V-ATPase activity is unclear.^16^^,^^17^ Altered V-ATPase expression has been reported in some DD patients,^18^ but not in ClC-5 KO mouse models.^11^ Accumulated work on ClC family members that function as ion exchangers (ClC-3, ClC-4, ClC-5, and ClC-7) increasingly suggests that the expulsion of one proton in exchange for two chloride anions by ClC-5 is necessary to maintain continued import of protons by the V-ATPase^17^ (Figure 1). In a recent study, Chang et al. demonstrated the importance of functional coupling between ClC-5 and V-ATPase for proper endosomal acidification and maturation.^19^ Reduced Cl^−^/H^+^ transport stoichiometry was observed in three different ClC-5 disease-causing variants (S244L, R345W, and Q629*) when expressed in *Xenopus laevis* oocytes. Additionally, due to its voltage-gating, ClC-5 requires sufficient accumulation of protons within endosomes to be activated. This suggests that some level of acidification is necessary for ClC-5 activation, and converselythat ClC-5 activity is required for additional acidification.

Progressive luminal acidification of endosomes is well known to be critical for proper sorting and function along the endolysosomal pathway, and *in vitro* studies using acridine orange trapping to quantify acidification found that endocytic vesicles isolated from ClC-5-deficient mouse kidneys acidify more slowly and less efficiently than their wild-type (WT) counterparts.^13^ However, the observation that some pathogenic mutations do not necessarily result in impaired endosomal acidification has led to the suggestion that regulation of endosomal [Cl^−^] rather than pH may be the primary function of ClC-5. In an early test of this hypothesis, Novarino et al. generated a knock-in mouse model with a novel mutation in ClC-5 that uncouples Cl^−^/H^+^ transport (E211A), rendering the mutant a Cl^−^ channel.^20^ These mice presented with the same renal phenotype observed in ClC-5 KO mice, including reduced uptake of dextran and lactoglobulin as well as reduced expression of megalin and cubilin in the PT. However, renal cortical vesicles isolated from these mice had normal rates of endosomal acidification.^20^ These studies were interpreted to suggest that impaired endosomal acidification was insufficient to explain the DD phenotype. It is worth noting that because *in vitro* acidification assays are performed using nonphysiologic conditions and because endosomal pH *in vivo* is not easily measured, the uncoupled mutant may in fact be partially defective at regulating endosomal acidification in the PT. A more recent study examined a similar disease-causing mutation (E211G), which like E211A functions as a Cl^−^ channel rather than as a H^+^/Cl^−^ exchanger.^21^ When transfected into HEK293T cells, the mutant ClC-5 trafficked normally and did not impair endosomal acidification. However, because these cells retain their normally functioning endogenous acidification machinery, the conclusions of this experiment regarding the role of ClC-5 in endosomal ion homeostasis are limited. In a third study, conditionally immortalized human PT cells (ciPTECs) isolated from the urine of three DD patients, each with a different mutation (30:insH, del132-241, and R637X), were characterized to examine the link between acidification and ClC-5 function.^22^ Whereas all three cell lines exhibited reduced endocytic uptake of albumin and transferrin compared with control ciPTECs, increased endosomal pH was observed in only two of the three patient cell lines (del132-241 and R637X). One of the two mutants (del132-241) with defective endosomal acidification also exhibited reduced uptake of a fluid-phase marker. Whether these differences in endosomal capacity are solely dependent on ClC-5 status or reflect clonal differences between the independently isolated cell lines is unknown. Nevertheless, this study further supports the possibility that altered endosomal acidification is not essential for development of the DD phenotype and remains one of the few studies of ClC-5 function in a representative human kidney cell line.

These recent studies suggest that the coupling of H^+^ and Cl^−^ transport in early endosomes is a critical function of ClC-5, and that [Cl^−^] as well as [H^+^] concentration may be an important factor in endosome maturation. In normal endosome maturation, the decrease in pH within endosomes is necessary for ligand dissociation, sorting, and degradation. Chloride concentration increases as endosomes mature,^23^ but its role in the regulation of endosome maturation and recycling is unclear.^24^ If endosome maturation is impaired in DD, either through increased pH or decreased [Cl^−^], a reduction in lysosomes and/or an increase in number and size of endosomes would be predicted. To date, no major structural differences have been reported in the endolysosomal pathway of Dent patients or mouse models.^11^^,^^18^ Nonetheless, there is evidence that lysosomal function may be affected. Reduced levels of lysosomal enzymes B-hexosaminidase and cathepsin B were reported in ClC-5 KO mice.^12^ This is suggestive of a degradative defect upon the loss of ClC-5, which could reflect inefficient routing of newly synthesized hydrolases to lysosomes. These enzymes normally bind to mannose 6-phosphate receptors in a pH-dependent manger in the *trans*-Golgi network, and dissociate upon reaching more acidic late endosomes for their ultimate delivery to lysosomes.^25^ When acidification is impaired, the hydrolases are instead released into the extracellular fluid. Moreover, the defect in lysosomal composition may be further exacerbated by the loss of megalin, which normally recovers filtered and/or secreted lysosomal enzymes from the tubule lumen to help maintain PT lysosomal function.^26^

The two cytosolic CBS (cystathionine β-synthase) domains at the C-terminus of ClC-5 form a nucleotide-binding pocket that binds to, but does not hydrolyze, ATP and ADP. The binding of ATP to the two CBS domains enhances ClC-5 activity^27^ and induces a conformational change in the C-terminal region.^28^ Recently, Grieschat et al.^29^ showed that when expressed in HEK293 cells, ClC-5 transport activity increases in response to available ATP and ADP and decreases when AMP levels are elevated. This suggests that ATP levels may modulate the functional coupling of ClC-5 and V-ATPase (which itself requires a supply of ATP for its activity) to acidify endosomes. Several mutations in the CBS domains have been identified in DD patients.^8^ The role of ATP binding to ClC-5 in DD pathology is unclear, but ClC-5 may act as metabolic sensor that tunes V-ATPase’s ability to acidify endosomes in response to the availability of ATP and ADP. ATP binding may also be important for proper folding of ClC-5 since multiple pathogenic mutations in the CBS domains cause ER retention.^22^^,^^28^

Regardless of whether Cl^−^ or H^+^ is the primary mediator of ClC-5 endosomal function, it is clear that many disease-causing mutants do alter endosomal acidification *in vivo*. Because impaired acidification of endocytic pathway compartments is well known to impair lysosomal delivery and inhibit protein degradation, this raises a conundrum—Why are megalin and cubilin protein levels reduced in DD PTs? An ancillary question is why disease-causing uncoupling mutations that may not impact pH apparently have similar effects on steady-state receptor levels. In addition, knowing pH and [Cl^−^] of endosomes *in vivo* may provide better correlation of genotype to phenotype. Resolving these questions will require a better understanding of the uniquely specialized endocytic pathway of the PT and how it is regulated.

## Role of ClC-5 in Megalin and Cubilin Receptor Trafficking

Reduced uptake of filtered proteins and expression of endocytic receptors are hallmarks of DD. The drop in expression of megalin and cubilin protein levels in DD mouse models occurs without corresponding changes in mRNA,^12^ which suggests that loss of ClC-5 shortens the half-life of these receptors. Further evidence for the regulatory role of ClC-5 in the endocytic pathway is demonstrated by a recent study in which ClC-5 KO male mice transplanted with WT bone marrow (BM) had increased levels of ClC-5 and megalin in PT cells and reduced severity of LMW proteinuria, ostensibly because of vesicular complementation of ClC-5 from engrafted BM-derived cells to PT cells via tunneling nanotubes.^30^ Understanding how membrane traffic is impacted to cause the reduction in megalin and cubilin levels is challenging because we lack a detailed model for protein sorting along the PT apical endocytic pathway. Moreover, recent studies suggesting axial differences in apical membrane uptake along the length of the PT further complicate the development of an integrated model for how proteins and other molecules are efficiently recovered from the kidney ultrafiltrate. Specific questions related to DD that will be discussed below include—Does loss of megalin in DD affect the integrity of the endocytic pathway? Do changes in cubilin expression, distribution, and trafficking in DD parallel those described for megalin? What step(s) in trafficking are affected in DD to cause the observed reduction in receptor levels? And does DD differentially affect the uptake of filtered ligands in the different nephron segments (S1, S2, and S3) that comprise the PT?

### The Integrity of the Endocytic Pathway in DD

Both imaging and biochemical data support the loss of megalin and cubilin receptor expression in DD animal and cell models. The difference between megalin and cubilin expression in ClC-5 KO versus WT cells is most readily obvious in PTs of heterozygous female mice.^10^^,^^20^ Due to random X-inactivation, these tubules have neighboring regions of cells that express or lack ClC-5, and allow direct estimation of the role of ClC-5 in the expression of megalin and cubilin within the same PT. By western blotting, megalin and cubilin protein levels in ClC-5 KO kidneys were measured to be 72% and 26%, respectively, of those quantified in WT mice.^12^ Additionally, subcellular fractionation revealed a shift of megalin and cubilin from the brush border to endosomes in ClC-5 KO mice compared with WT mice. This analysis could not distinguish between AEEs, AVs, or DATs, but it represents the only quantitative data available on the expression and distribution of megalin and cubilin following loss of ClC-5.

The relatively modest (∼30%) reduction in megalin levels observed by blotting belies the dramatic reduction observed by immunostaining. Resolving the discrepancy is important, because megalin expression has an essential function in maintaining the integrity of the apical endocytic pathway *in vivo*. Ultrastructural studies of PTs in megalin KO mice and in kidney biopsies from Donnai–Barrow syndrome patients with megalin mutations reveal a striking reduction in the density of coated pits, early endosomes, and lysosomes.^31–33^ No noticeable major ultrastructural changes in the endocytic pathway in DD mouse models^12^ or patients^18^ have been observed, suggesting that the loss of megalin protein in ClC-5 KO cells is less extreme than that suggested in fluorescence images of heterozygote PTs. Christensen et al. also found unchanged expression by western blotting of endosomal markers Rab5, Rab7, and Rab4b total mouse kidney lysates.^12^ However, one study did observe reduced number of early endocytic compartments in a DD mouse model.^11^ It is possible that there is a threshold level of megalin required to maintain the highly elaborated PT apical endocytic pathway, and that a specific range in the reduction in megalin levels observed in DD results in variable changes to this pathway. More detailed assessments of endosomal compartment abundance and size in normal and DD PTs, and correlation of megalin expression levels with these measurements, will help resolve this issue.

In addition to its global function in maintaining the PT apical endocytic pathway, recent studies suggest that megalin has distinct roles in regulating endocytic function in different PT nephron segments.^34^^,^^35^ While loss of megalin clearly impairs endocytic uptake along the entire PT, a reduction in megalin expression may have different effects on endocytic pathway integrity within each subregion of this nephron segment (S1, S2, S3). Indeed, the evidence for megalin-dependent elaboration of the endocytic pathway is based on ultrastructural examination of a limited sample of undefined PT nephron segments. More thorough analysis of the spatial distribution of endosomal compartments in distinct PT segments is needed to determine whether the integrity and overall capacity of the apical endocytic pathway are altered in DD.

### The Role of Cubilin in DD

Although most studies have monitored changes in megalin expression in DD, albumin and many other filtered ligands bind to cubilin rather than megalin.^35^^,^^36^ Cubilin lacks a cytoplasmic domain and relies on association with the transmembrane protein amnionless for delivery to the plasma membrane.^37^ Additionally, cubilin binds to megalin, and the two receptors have been generally assumed to traffic together as a complex. However, the relative abundance of megalin and cubilin varies dis-coordinately in S1, S2, and S3 PT segments, raising the possibility that megalin and cubilin can function independently of each other.^34^^,^^38^^,^^39^ Moreover, as noted above, western blotting suggested a much greater reduction in cubilin receptor levels in DD mice compared with megalin.^12^ Currently, we have no quantitative assessment of the relative rates of internalization or recycling for these receptors or how they function synergistically to maintain efficient ligand uptake in the PT. We need a better understanding of how the dis-coordinate changes reported in steady-state levels of cubilin versus megalin contribute to the impaired uptake of filtered ligands in DD.

### Trafficking Steps Affected in DD

**Figure 2.**
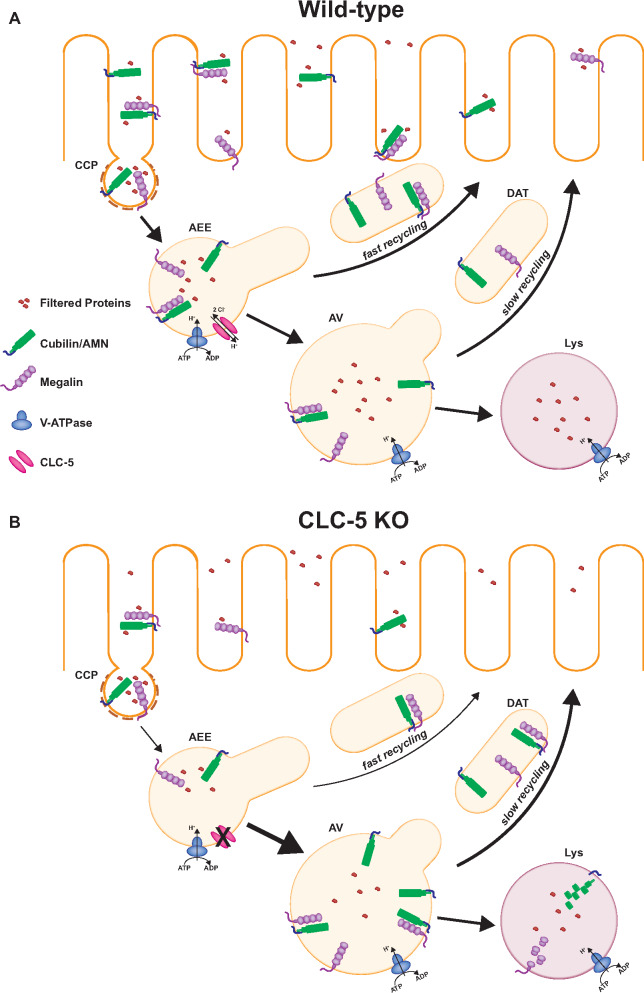
Trafficking Through the Apical Endocytic Pathway in Normal and ClC-5 KO PT Cells. (**A**) Megalin and cubilin/amnionless (AMN) receptors at the apical plasma membrane of PTs bind to and internalize filtered ligands via CCPs. After uncoating, CCPs fuse with apical early endosomes (AEEs) which mature into AVs. Acidification of early endosomes causes ligand dissociation from receptors. Receptors recycle to the apical membrane via DATs that emerge from AEEs (fast recycling) and AVs (slow recycling). Soluble ligands remain in maturing endosomal compartments and are ultimately delivered to lysosomes (Lys) for degradation. (**B**) Loss of ClC-5 function results in reduced levels of steady-state megalin and cubilin and their redistribution from the apical membrane into intracellular compartments. These changes may reflect reduced receptor recycling and increased shunting to degradative compartments as well as a reduced endocytic rate as noted by the arrow thickness.

In addition to a reduction in the total number of receptors, the distribution of megalin and cubilin appears to be altered in ClC-5 KO mice. Christensen et al. found reduced expression of megalin and cubilin in brush border relative to endosomal membranes.^12^ A disruption in the efficiency of endosome maturation could reduce the recycling rate of megalin and cubilin which would result in fewer available receptors at the surface and decrease the overall endocytic capacity of the proximal tubule (Figure 2). Since ClC-5 is primarily localized to AEEs, it is conceivable that the fast recycling pathway might be primarily affected. If a smaller fraction of receptors is recycled in DD, a larger number of receptors could be shunted away from the recycling pathway and toward degradative routes. This could lead to a reduction in receptor half-life and consequently to lower steady-state levels of megalin and cubilin, assuming that degradation in lysosomes is not itself affected by improper endosomal acidification and maturation. Importantly, because megalin and cubilin have relatively long half-lives and cycle rapidly, only a small change in sorting efficiency integrated over many rounds of recycling could lead to the dramatic reductions in receptor levels observed. This may also explain why no effects on receptor trafficking rates have been reported. Moreover, in addition to its role in maintaining endosomal ion homeostasis, ClC-5 may also modulate endocytic function via other mechanisms. For example, ClC-5 has been shown to interact with the actin-binding protein aspartyl aminopeptidase (DNPEP) as well as with megalin, cofilin, and NHERF2.^40–42^ These interactions may facilitate megalin internalization or modulate cytoskeletal dynamics.

In order to pinpoint how loss of ClC-5 affects endocytic receptor levels, we need to know the endocytosis, recycling, and degradation rates of megalin and cubilin in WT and ClC-5 KO cells. These rates, combined with quantitative assessment of megalin and cubilin expression levels and their distribution in PT cells, including the fraction at the surface, within AEEs, and within AVs will provide insight into whether the endosome maturation and/or degradation rates are affected. Using these aggregate data to develop mathematical models of megalin and cubilin trafficking will enable identification of which rates are altered in DD. The incorporation of individual rate constants with data from steady-state distribution and protein level measurements into a kinetic model makes this a powerful approach to identify small differences in the kinetics or sorting efficiency within a single trafficking step that result in a large effect in receptor expression over many cycles. Ideally, a detailed model would further enable distinction between slow and fast recycling pathways to determine if one or both are affected in DD.

### Recovery of Filtered Proteins along the Length of the PT

The trafficking defect in cells lacking ClC-5 is presumably not limited to megalin and cubilin, and indeed, altered trafficking of other PT apical membrane proteins including NHE3 and NaPi-2 has been reported.^10^^,^^43^ Additionally, some studies have found that fluid-phase uptake by PT cells is also affected upon loss of ClC-5. Uptake of dextran is reduced in cells expressing some pathogenic ClC-5 mutants^22^ and in ClC-5 KO mice^10^ as well as in knock-in mice expressing the ClC-5 E221A uncoupling mutant.^20^ Fluid-phase uptake of filtered proteins may play a larger role in the later segments of the PT, as the robust water and ion reabsorption in the PT S1 segment progressively concentrates any remaining molecules in the ultrafiltrate. The S2 segment expresses more megalin mRNA and protein than S1, but less cubilin, and this segment may mediate cubilin-independent uptake of filtered ligands and other molecules in the fluid phase.^34^^,^^38^^,^^44^ Thus, a thorough analysis of the endocytic pathway along the length of the PT is needed to understand how the uptake of normally filtered proteins is impacted by the loss of ClC-5.

## Possible Mechanisms for Impaired Receptor Trafficking in DD

Alterations in endosomal [H^+^] and other ions impact myriad downstream pathways, and the molecular mechanism by which loss of ClC-5 activity translates into reduced megalin and cubilin expression remains elusive. Additionally, specific DD-causing mutations may differentially affect downstream steps, contributing to phenotypic heterogeneity in cellular responses and disease severity.^9^^,^^22^ Determining the specific trafficking pathway(s) impacted in DD is a critical step in identifying potential pathways affected and possible targets for therapy.

An unexplored mechanism by which loss of ClC-5 might alter membrane traffic that deserves consideration is via effects on lipid composition or dynamics. In support of this possibility, a microarray study comparing gene expression in dissected PTs of ClC-5 KO and WT mice reported that the majority of pathways significantly affected were related to lipid metabolism, and especially to fatty acid and cholesterol metabolism.^45^ While transcriptional changes in lipid metabolism genes have not been confirmed in DD patients and the lipid content and composition of the kidney cortex in ClC-5 KO mice or DD patients have yet to be quantified, we have observed changes in the cellular distribution of the cholesterol marker filipin in PTs isolated from ClC-5 KO male mice and heterozygous female mice when compared with WT controls (unpublished data).

**Figure 3.**
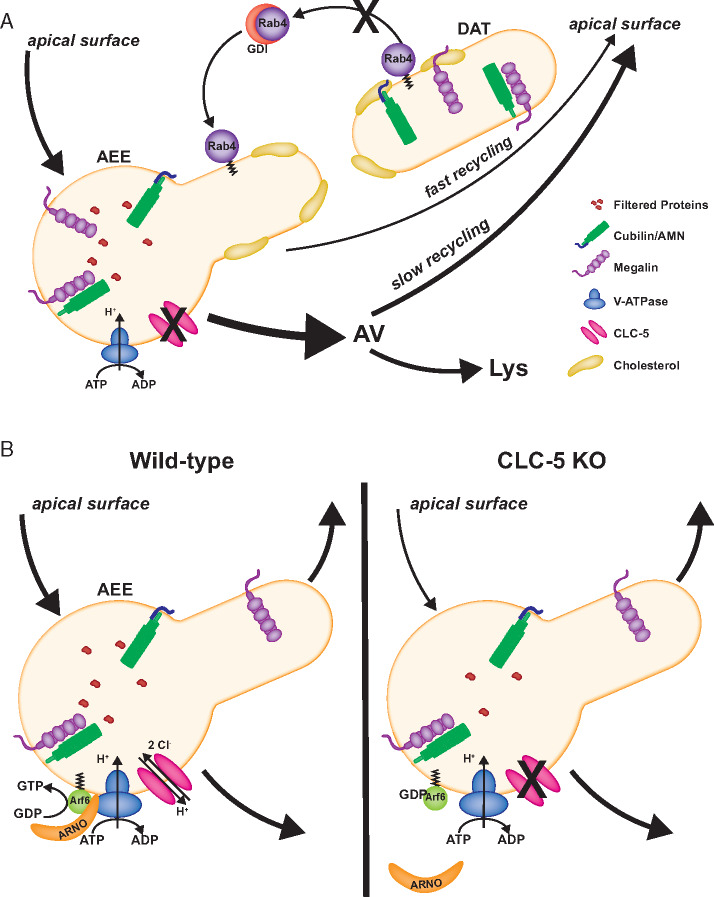
Potential Mechanisms for Impaired Endocytic Function in DD. (**A**) Impaired ion homeostasis due to loss of ClC-5 may result in the accumulation of cholesterol in early endosomes. Previous studies show that excess endosomal cholesterol prevents efficient removal of Rab4 by the GDI from target membranes for reactivation. As a consequence, engagement of the slow recycling pathway is enhanced. In DD, increased intracellular dwell time and/or transit of megalin and cubilin/AMN through AVs may result in their enhanced delivery to lysosomes, resulting in a steady-state redistribution of these receptors to intracellular compartments and their more rapid degradation, consistent with observations in ClC-5 KO mice (see Figure 2B). (**B**) Alternatively, or in addition, loss of ClC-5 may impair V-ATPase activity, leading to reduced activation of Arf6 by its GTP exchange factor ARNO. Knockdown studies show that this blocks traffic from early to late endosomes, resulting in protein accumulation in early endosome and subsequent inhibition of endocytosis (see text).

Modulation of cellular lipid composition, and of cholesterol in particular, is known to impact endosomal recycling via several mechanisms. For example, Choudhury et al. showed that accumulation of cholesterol in Niemann–Pick cells impaired Rab4-dependent fast recycling, resulting in increased recycling via the slower Rab11-dependent pathway.^46^ Whereas Rab4 expression was not altered, its activation appeared to be selectively impaired relative to other Rab proteins due to its reduced extraction from endosomal membranes by GDP Dissociation Inhibitor (GDI), a protein that helps return GDP-bound Rabs to their original site of action to be reactivated (Figure 3A). Impaired fast recycling in DD due to the accumulation of cholesterol in AEEs could explain the intracellular redistribution of megalin and cubilin observed by cell fractionation.^12^ Reduced fast recycling efficiency of these receptors and/or their prolonged intracellular retention could increase their routing to lysosomal compartments.

How the absence of ClC-5 might result in alterations to cholesterol distribution and/or metabolism remains unclear. Loss of ClC-5 activity clearly affects the ion composition of endosomes, and endosomal Ca^2+^ is a known regulator of endosomal cholesterol content.^47^ It is also possible that any changes in cholesterol are a secondary consequence of the reduction in megalin and cubilin levels in DD. Megalin and cubilin bind to and internalize filtered apolipoproteins, high-density lipoproteins, and fatty-acid bound proteins and play a large role in the lipid metabolism of PT cells. Like ClC-5 KO mice, megalin KO mice also have apparent alterations in lipid metabolism.^48^ The reduction in expression of these receptors in DD may further exacerbate or counteract any direct effect on lipid metabolism due to loss of ClC-5.

Alternatively (or in addition), ClC-5 may influence endosomal cholesterol levels through the ARNO/Arf6 pathway (Figure 3B). Arf6 is a small GTPase whose activity is regulated by the GTP exchange factor ARNO. In the PT, these proteins interact with V-ATPase on AEEs.^49^ Intriguingly, this interaction is dependent on endosomal acidification, and disruption of this interaction reduced uptake of fluorescent albumin.^50^^,^^51^ Recently, Marquer et al. demonstrated that KO of Arf6 in mouse embryonic fibroblasts results in the accumulation of phosphatidylinositol 4-phosphate in early endosomes causing mistrafficking of proteins and the accumulation of free cholesterol in late, but not early, endosomes.^52^ Impaired endosomal acidification in DD could reduce ARNO/Arf6 activity resulting in impaired trafficking and altered cholesterol distribution. An accumulation of cholesterol in late endosomes could alter trafficking from AVs and/or affect lysosomal function to impact megalin and cubilin levels. Whether impaired ARNO-mediated activation of Arf6 contributes to the pathogenesis in DD could be tested in knock-in cells expressing an uncoupled ClC-5 mutant or in the subset of patient cell lines where endosomal [Cl^−^] may be altered in the absence of a change in pH.^20^^,^^22^ The specific distribution of cholesterol among endosomal compartments in cells lacking functional ClC-5 also needs to be quantified. This would potentially indicate through which pathway cholesterol metabolism is altered in DD and whether it plays a role in the reduced expression of megalin and cubilin.

## Summary and Conclusions

After considerable study, we are beginning to understand how ClC-5 functions within endosomes to contribute to [H^+^] and [Cl^−^] levels. Whether improper acidification is key to dysfunction in DD remains unclear. Continued optimization of fluorescent probes to quantify organelle ion composition will facilitate a better understanding of the role of ClC-5 in its native tissues. Additionally, the downstream consequences of impaired ion homeostasis that lead to LMW proteinuria are unresolved. The loss of ClC-5 leads to reduced uptake of filtered proteins that likely reflects the reduced expression of megalin and cubilin observed in DD models. How ion homeostasis is linked to this outcome is the next major question to tackle. Deciphering these mechanisms will require an experimental shift toward cell biological and computational approaches. Specifically, a better appreciation of the trafficking pathway and kinetics of megalin and cubilin in normal and DD PTs will provide much needed insight into the role of ClC-5 in regulating endosome maturation and trafficking. The availability of improved PT cell models that better approximate endocytic function of the PT *in vivo* will facilitate this effort together with advanced gene-editing technologies to introduce pathogenic mutations in ClC-5.^53^ Expansion of these studies into a comprehensive model that integrates differences in expression levels of receptors and the role of fluid-phase uptake along the length of the PT is essential for understanding how filtered ligands are handled in the absence of ClC-5 function. In this regard, progress deciphering the pathogenesis of DD will likely inform our understanding of other conditions that result in LMW proteinuria and may suggest global therapeutic targets to modulate PT endocytic capacity.
